# Supplementary figures and images for: Delineating the functional activity of antibodies with cross-reactivity to SARS-CoV-2, SARS-CoV-1 and related sarbecoviruses
Source: PLoS Pathog. 2024 Oct 28;20(10):e1012650. doi: 10.1371/journal.ppat.1012650 (PMC11542851; doi:10.1371/journal.ppat.1012650)

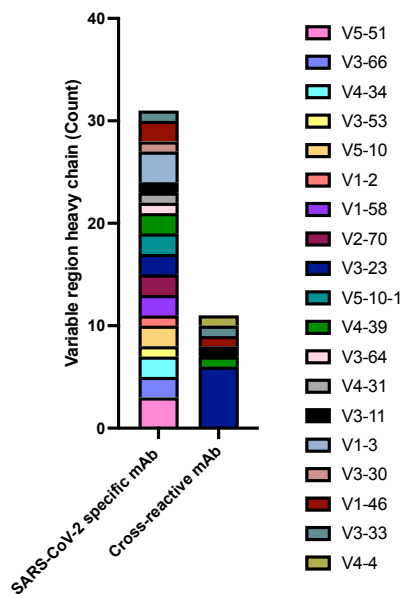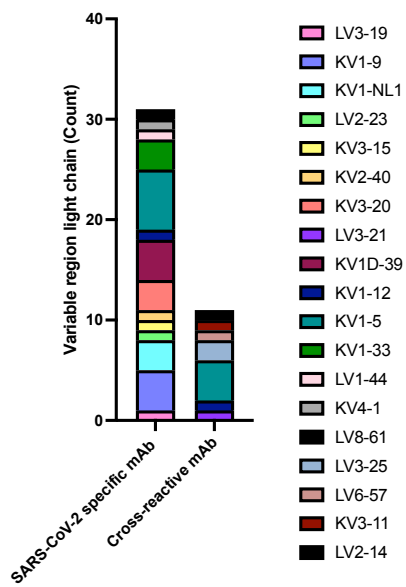

Supplement: S1 Fig — Counts for the heavy and light chain V-gene segments among C68 RBD mAbs (related to Fig 1C) based on partis computational software analysis. Antibody heavy (left panel) and light (right panel) chain amino acid sequences found in S1 Table. (PDF) [file ppat.1012650.s001.pdf]

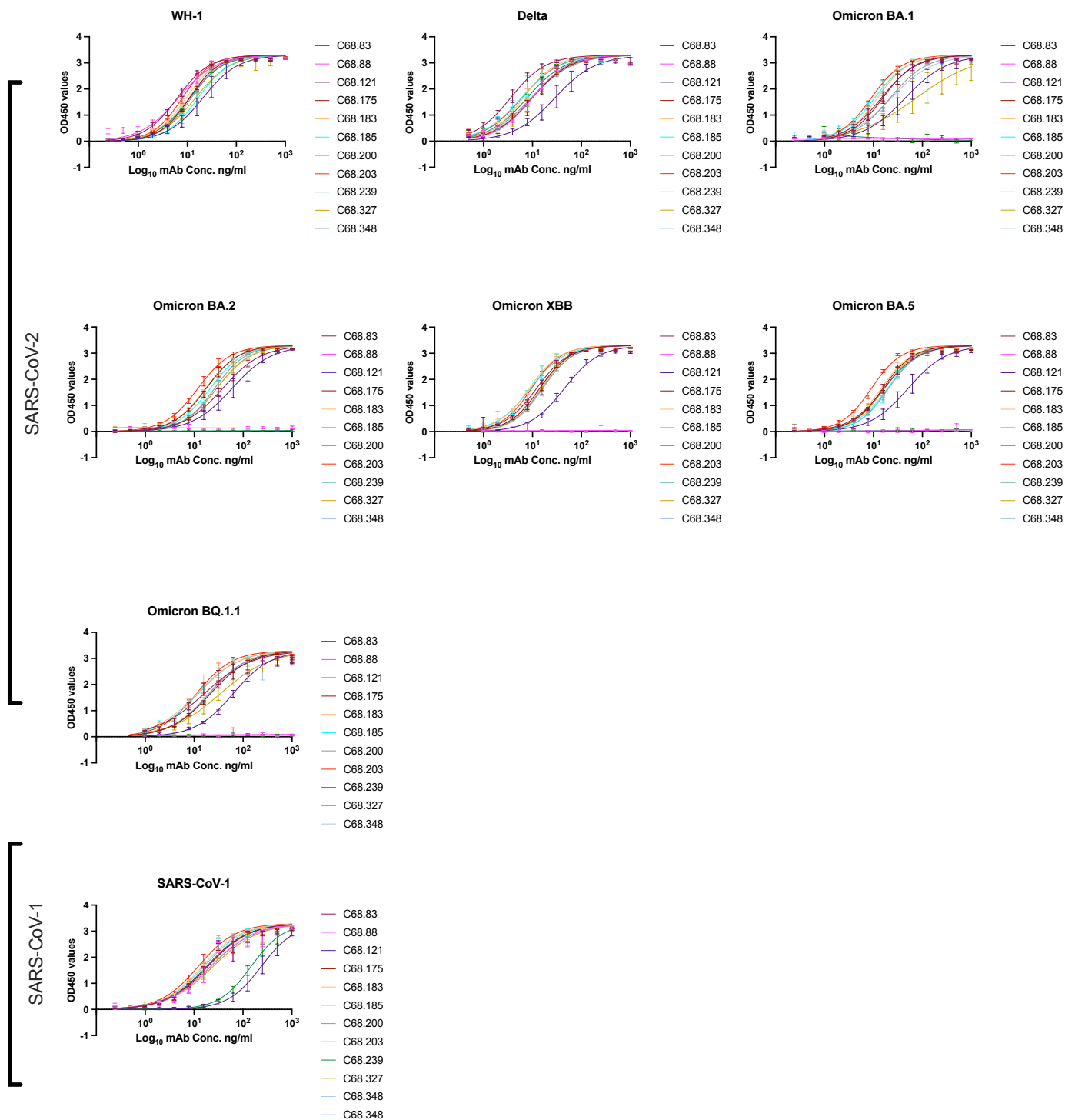

Supplement: S2 Fig — Binding of C68 mAbs to SARS-CoV-2 WH-1, SARS-CoV-2 variants (Delta, Omicron BA.1, Omicron BA.2, Omicron XBB, Omicron BA.4/BA.5, Omicron BQ.1.1) or SARS-CoV-1 recombinant spike trimers. The concentration of each mAb (ng/mL) is plotted on a log10 scale versus absorbance (OD450nm values). Half-maximal effective concentrations (EC50 values) calculated by nonlinear regression analysis from at least two independent technical replicates. Each mAb was serially diluted 2-fold for 11–12 total dilutions. Curves represent nonlinear regression fits with error bars that indicate SEM. (PDF) [file ppat.1012650.s002.pdf]

**A**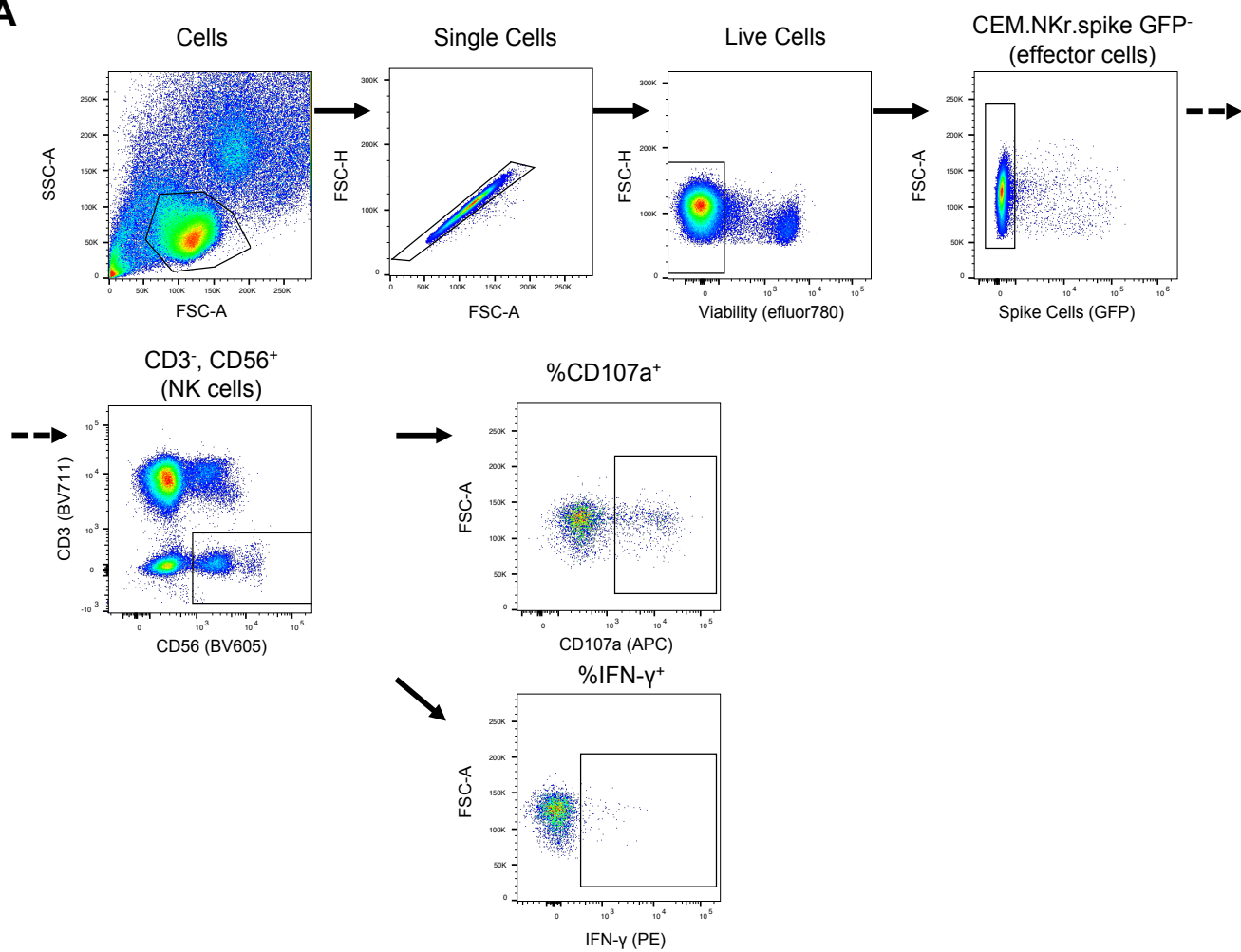**B**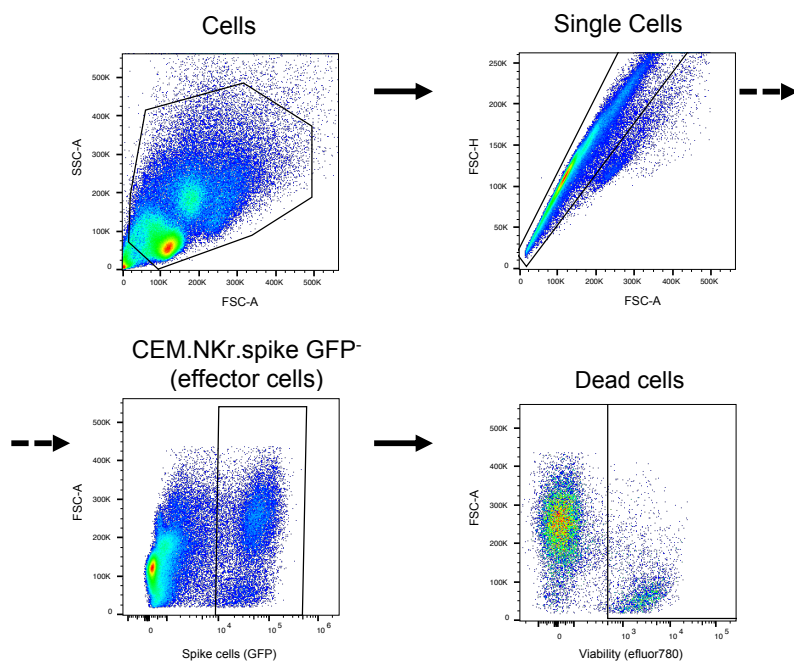

Supplement: S5 Fig — (A) The ability of candidate mAbs to trigger ADCC was determined by flow cytometry. NK cell (gated as live, single cells, CEM.NKr.spike-, CD3-, CD56+ cells) activation was measured by surface expression of CD107a (a proxy for degranulation) and intracellular IFN-γ [106]. (B) Representative gating strategy for CEM.NKr.spike cell death. (PDF) [file ppat.1012650.s005.pdf]

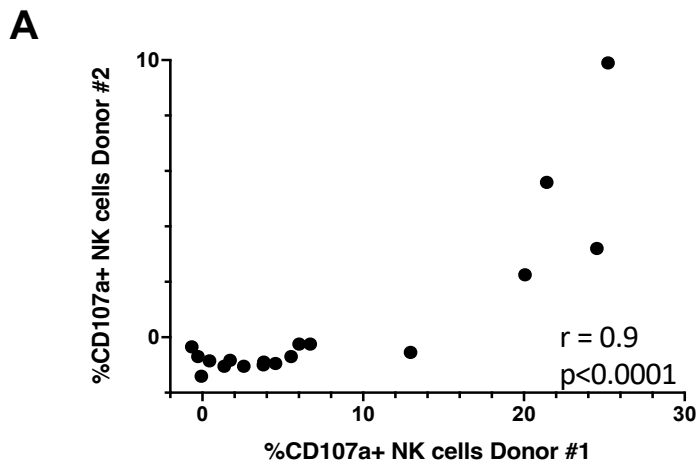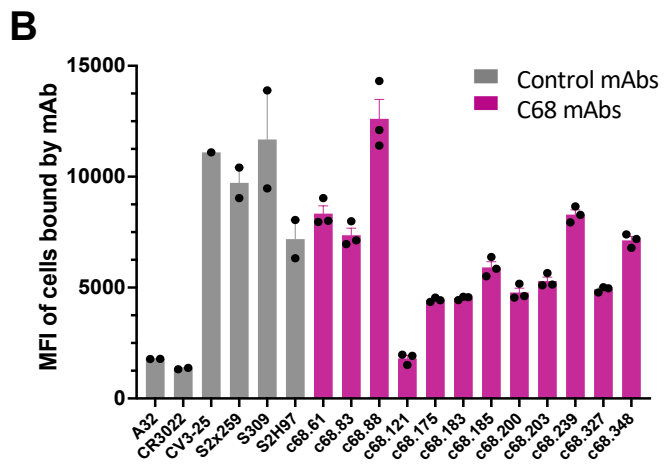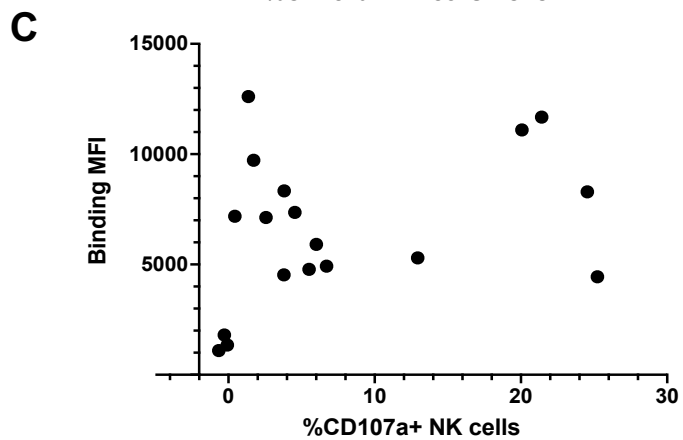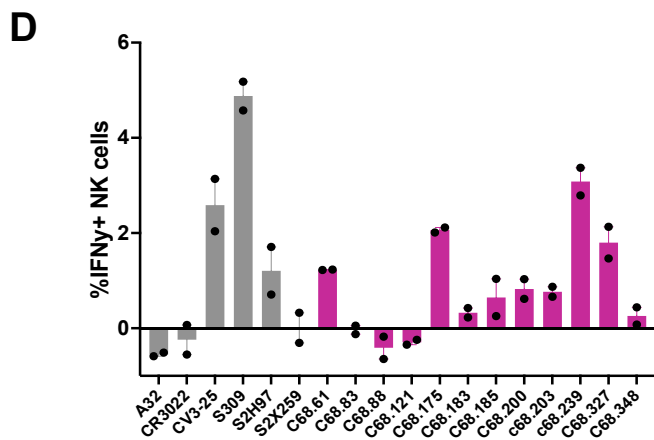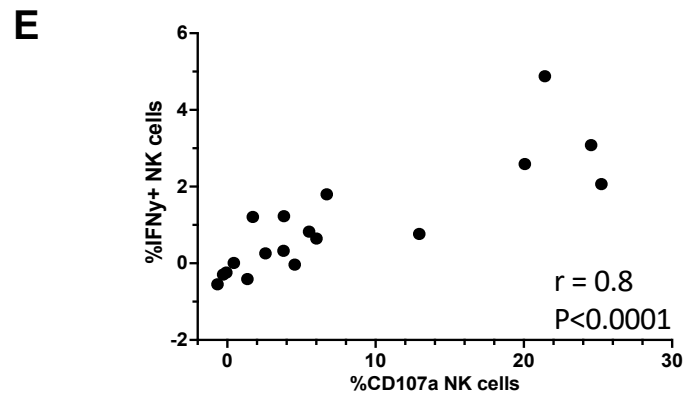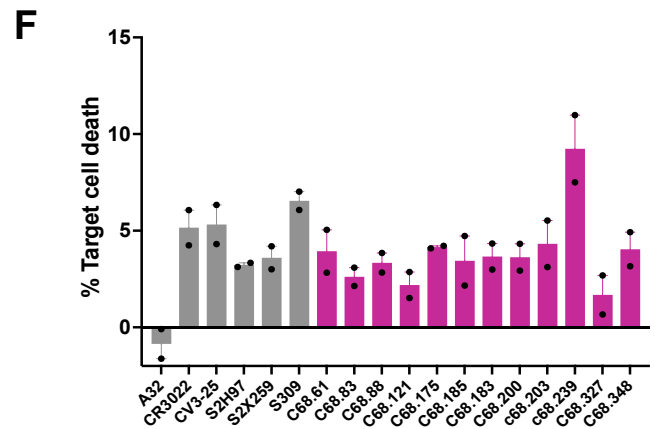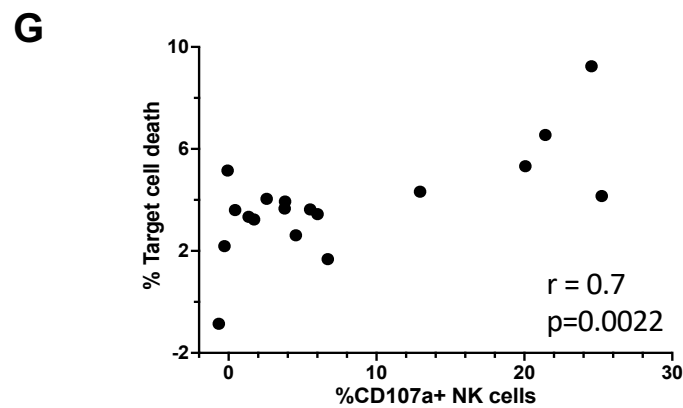

Supplement: S6 Fig — (A) Correlation plot of the ability of C68 mAbs and control mAbs (control mAbs: S309, CV3-25, A32, S2H97, S2H97) to trigger NK cell activation (%CD107a+) from two independent PBMC donors using Pearson’s correlation. (B) Measurement of the degree of binding to CEM.NKr cells expressing D614G SARS-CoV-2 spike (target cells). Control mAbs are shown in grey and C68 mAbs in pink. (C) Correlation plot between the degree of binding to target cells and the ability to trigger NK cell activation (%CD107a+). Pearson’s correlation analysis showed no association between NK cell activation (%CD107a+) and MFI (r = 0.3, p = 0.2). (D) Assessment of the ability of C68 mAbs to trigger NK cell activation (%intracellular IFN-γ). (E) Correlation between the ability of C68 mAbs to trigger NK cell activation as measured by percent cell surface CD107a or percent intracellular IFN-γ. (F) Percent of cell death within the target cell population. (G) Correlation plot between target cell death and %CD107a expression. Correlation plots between NK activation measurements (%CD107a, %intracellular IFN-γ, %target cell death) and binding of targets cells by mAbs (MFI) were assessed by Pearson correlation using PRISM. Results represent assays run in technical duplicate with background subtracted values, data shown from a single donor (for B-G). (PDF) [file ppat.1012650.s006.pdf]

A

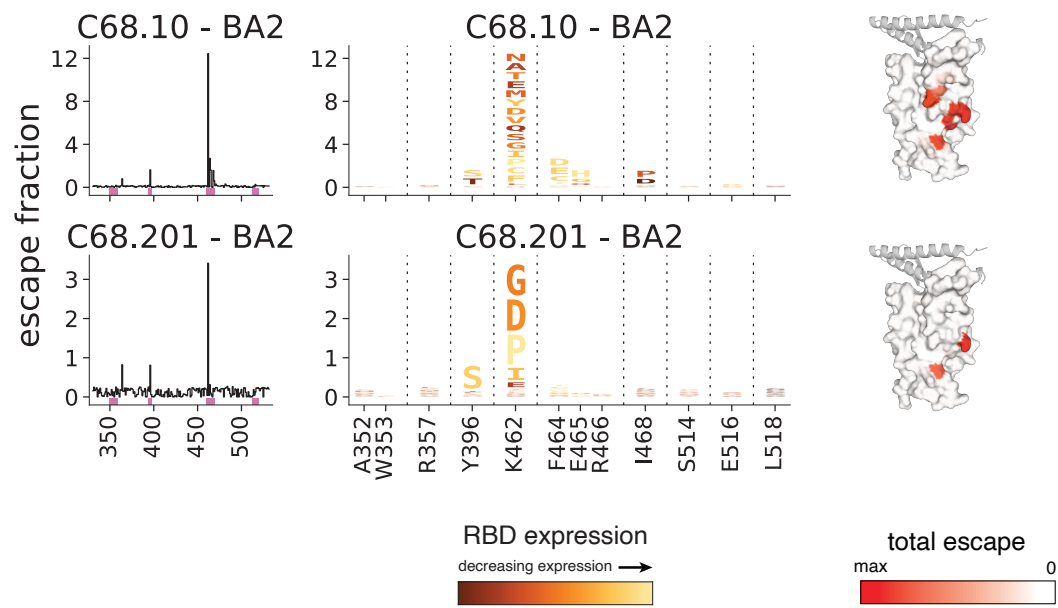

B

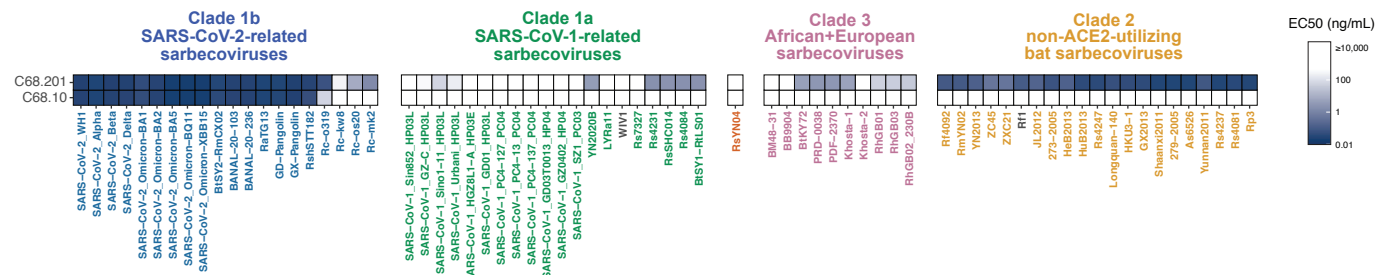

Supplement: S7 Fig — (A) Complete escape maps of antibody-binding escape mutations for C68 mAbs using a yeast-displayed SARS-CoV-2 RBD deep mutational scanning system (Omicron BA.2 RBD). Line plots (left panel) show escape at each site in RBD. Residue colors are assigned based on effect these mutations have on RBD expression (with yellow indicating mutations deleterious for RBD expression). The height of letters in the logo plots indicate level of escape by that amino acid at that site. Logo plot residue numbering is based on SARS-CoV-2 Omicron BA.2 (B) Pan-sarbecovirus RBD yeast display to assess C68 mAbs sarbecovirus RBD binding breadth. EC50 value represents the geometric mean across the independent barcodes for corresponding sarbecovirus RBD. (PDF) [file ppat.1012650.s007.pdf]

A

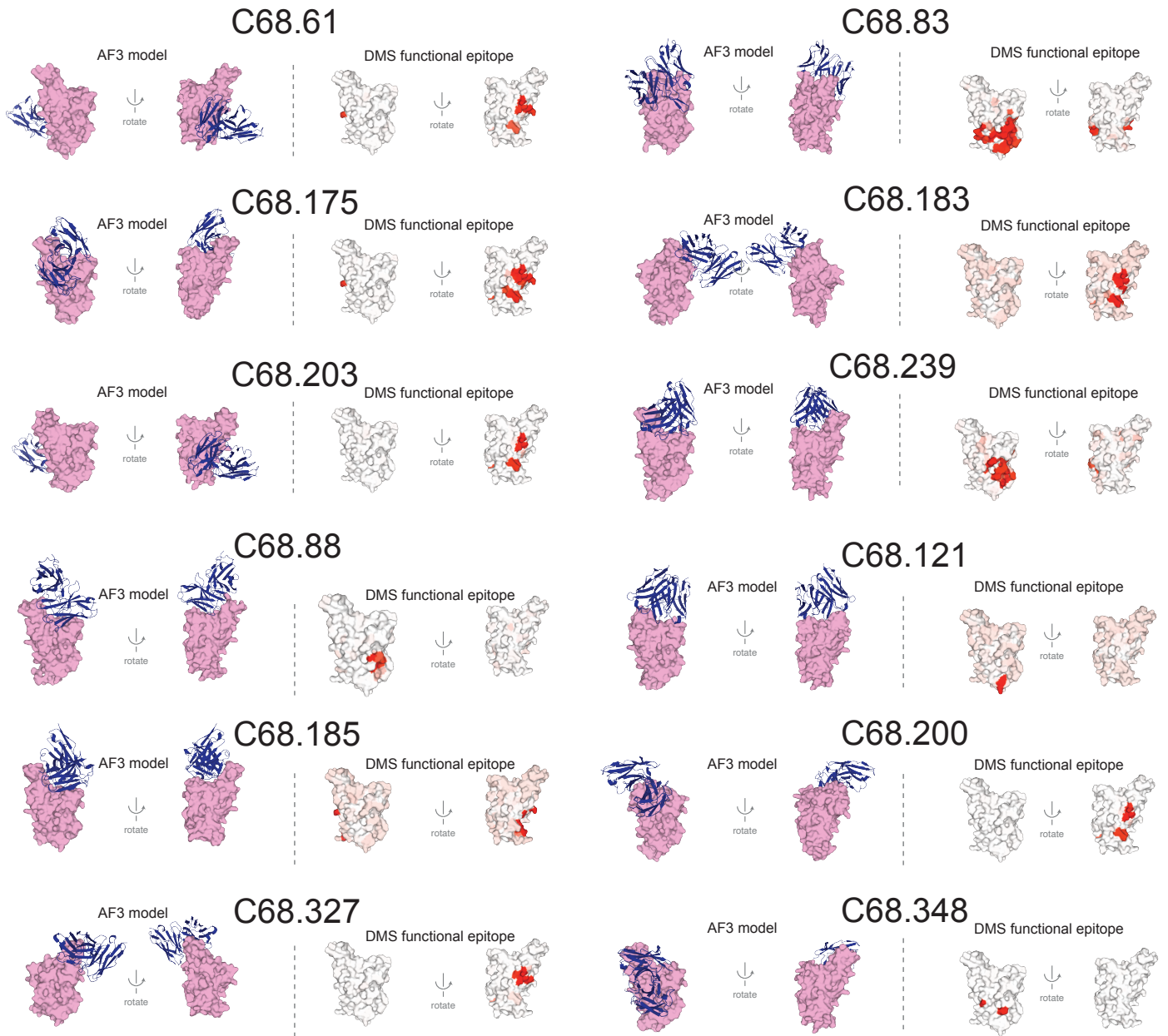

B

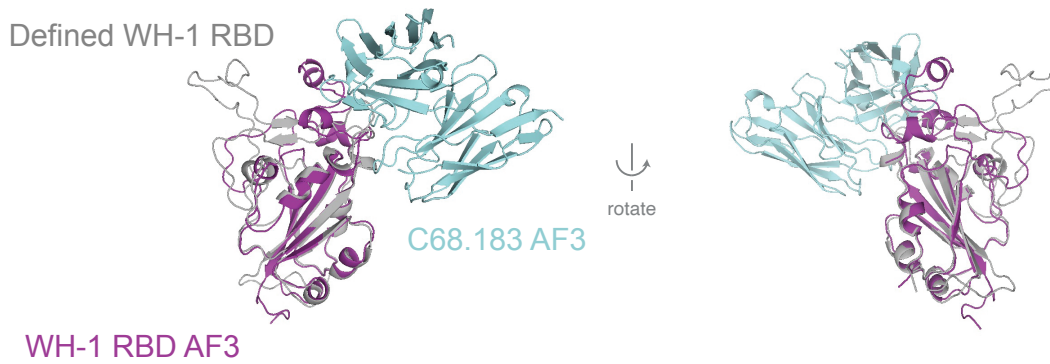

Supplement: S8 Fig — (A) Concordance between predicted AF3 and experimental DMS epitopes. For each antibody, views from two equivalent angles are shown for the predicted AF3 model on the left and the DMS epitope on the right (details as in Figs 3A and 4A). (B) Example of an improperly predicted RBD structure from the C68.183:RBD AF3 model. A structural alignment is shown between the experimentally determined RBD structure from PDB 6M0J (gray) and the predicted C68.183-bound RBD (purple), with predicted C68.183 chains shown in light blue, demonstrating accurate modeling of the RBD core but inaccurate modeling of the ACE2-contact loops. (PDF) [file ppat.1012650.s008.pdf]

**C68.61 escape mutations**

Genotype at S site 357, 464, 465, 466, 468

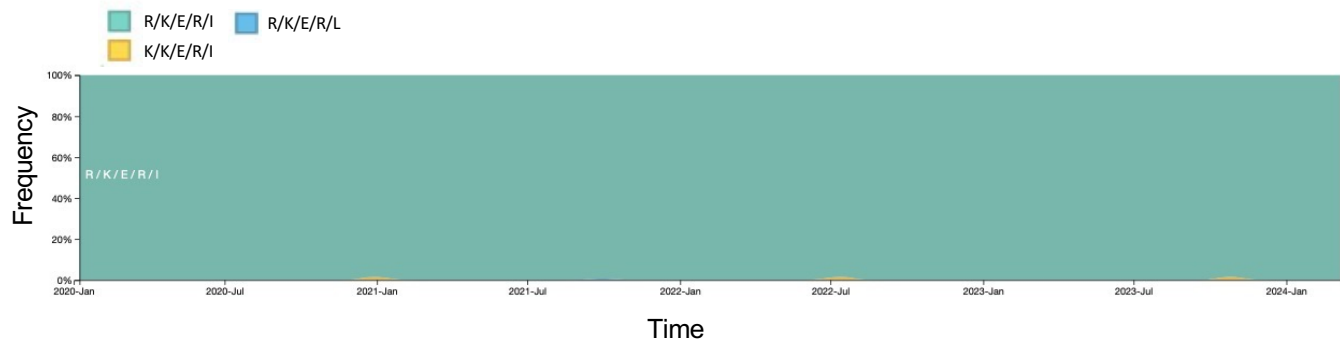

**C68.185 escape mutations**

Genotype at S site 396, 462, 464, 514, 516, 518

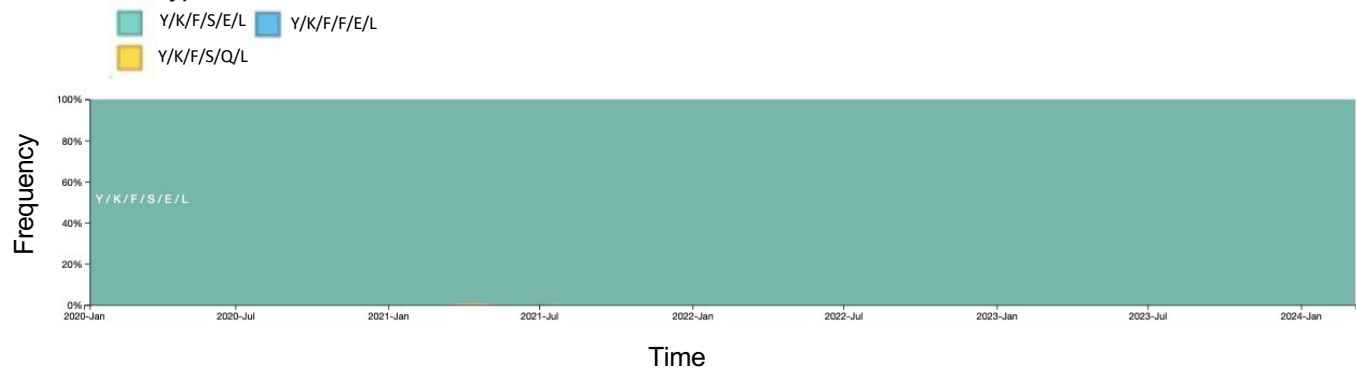

Supplement: S9 Fig — Area plot indicating the frequency of RBD binding escape mutants (from Fig 4A) found in SARS-CoV-2 sequences (Nextstrain) for C68.61 (top panel) and C68.185 (bottom panel) over time. Circles in teal are the residues found in the SARS-CoV-2 reference sequence (Wuhan-Hu-1) whereas mutations or deletions are indicated by yellow/blue circles. Depicts 3900 SARS-CoV-2 viral genomes sampled between Dec 2019 and Mar 2024 (S2 Table). (Nextstrain: https://nextstrain.org, CC-BY-4.0 license) (PDF) [file ppat.1012650.s009.pdf]

A

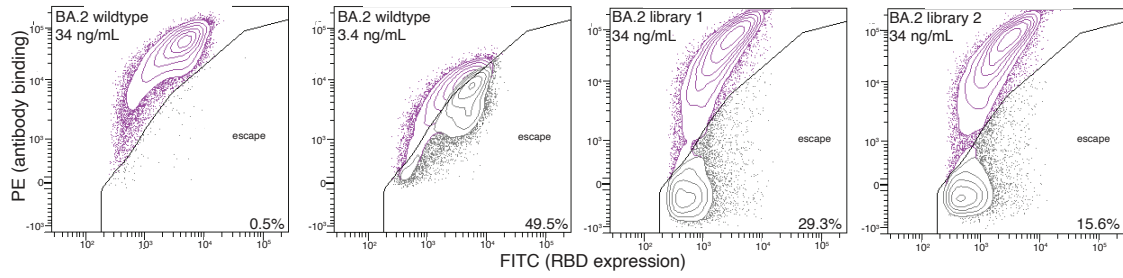

B

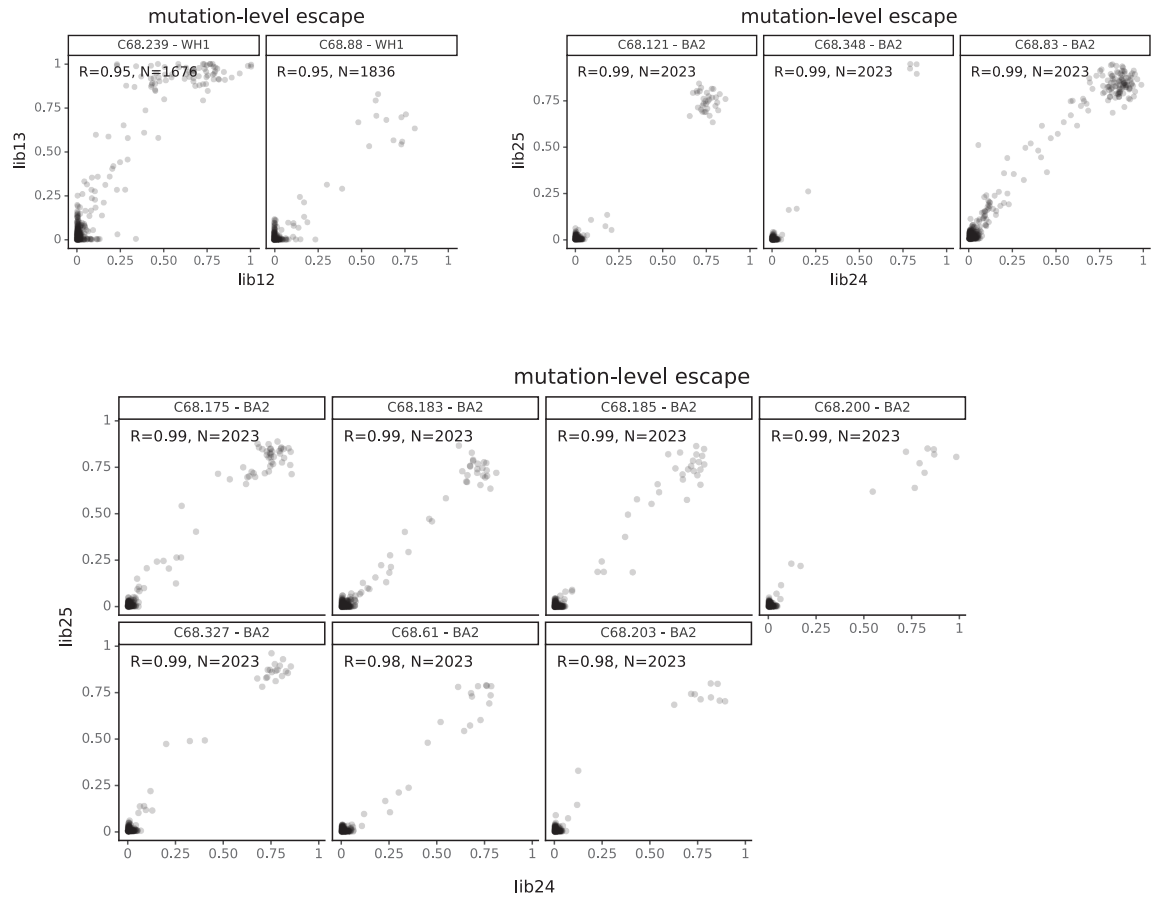

Supplement: S10 Fig — (A) Gating strategy to identify antibody escape mutants by gating on yeast mutants with reduced antibody binding. A yeast strain expressing the unmutated RBD of Wuhan-Hu-1 or Omicron BA.2 and flow cytometry were used to identify an EC90 and then yeast libraries were incubated with that EC90 concentration of mAb. Cells are incubated with PE-conjugated 1:200 goat anti-human-IgG and 1:100 FITC-conjugated chicken anti-Myc-tag. Cells are then gated on unmutated WH-1 or Omicron BA.2 control cells to identify escape mutants with >10x loss in antibody binding. (B) Correlation of mutation-level escape from independently generated mutant RBD libraries. (PDF) [file ppat.1012650.s010.pdf]
