# Supplementary material for: Delineating the functional activity of antibodies with cross-reactivity to SARS-CoV-2, SARS-CoV-1 and related sarbecoviruses
Source: PLoS Pathog. 2024 Oct 28;20(10):e1012650. doi: 10.1371/journal.ppat.1012650 (PMC11542851; doi:10.1371/journal.ppat.1012650)
Supplement: S3 Fig — (A) Sarbecovirus percent sequence identity to SARS-CoV-2 WH-1 RBD or SARS-CoV-1 RBD. (B) Geometric means (geomeans) of the IC50s and 95% confidence interval (95% CI) across SARS-CoV-2 variants only (left panel) and all tested sarbecoviruses (right panel) tested in Fig 2B. (PDF) [file ppat.1012650.s003.pdf]

A

	WH-1	Delta	Omicron BA.1	Omicron BA.2	Omicron XBB.1.5	Omicron BA.5	Omicron BQ.1.1	GD-Pangolin	SARS-CoV-1	RsSHC014	WIV1	LYRa3	Khosta-2	BtKY72
% Sequence identity to SARS-CoV-2 WH-1 RBD	-	99	93	92	89	92	90	96	74	77	77	75	68	73
% Sequence identity to SARS-CoV-1 RBD	74	75	72	71	71	71	72	75	-	82	96	95	70	74

B

Statistics for SARS-CoV-2 variants neutralization		
	Geomean	95% CI
C68.61	0.40	(0.25, 0.64)
C68.83	8.6	(2.2, 34)
C68.88	4.7	(0.47, 46)
C68.121	17	(12, 23)
C68.175	5.0	(1.7, 14)
C68.183	11	(4.6, 29)
C68.185	5.8	(2.6, 13)
C68.200	18	(13, 24)
C68.203	5.3	(1.6, 18)
C68.239	4.2	(0.35, 50)
C68.327	7.5	(3.4, 17)
C68.348	8.0	(2.2, 29)
S309	1.4	(0.3, 6.6)
CR3022	20	(20, 20)
S2H97	0.39	(0.17, 0.86)
S2X259	3.8	(0.43, 34)

Statistics for all sarbecovirus neutralization		
	Geomean	95% CI
C68.61	0.62	(0.24, 1.6)
C68.83	7.4	(3.2, 17)
C68.88	1.1	(0.19, 6.4)
C68.121	13	(8.4, 21)
C68.175	5.3	(1.9, 15)
C68.183	8.2	(3, 23)
C68.185	1.1	(0.26, 4.3)
C68.200	11	(4.4, 25)
C68.203	3.6	(1.3, 9.9)
C68.239	2.7	(0.52, 14)
C68.327	7.2	(2.9, 18)
C68.348	4.4	(1.6, 12)
S309	0.90	(0.23, 3.5)
CR3022	8.3	(3.5, 20)
S2H97	0.95	(0.32, 2.8)
S2X259	0.32	(0.05, 1.9)
