# Supplementary material for: Delineating the functional activity of antibodies with cross-reactivity to SARS-CoV-2, SARS-CoV-1 and related sarbecoviruses
Source: PLoS Pathog. 2024 Oct 28;20(10):e1012650. doi: 10.1371/journal.ppat.1012650 (PMC11542851; doi:10.1371/journal.ppat.1012650)
Supplement: S4 Fig — (A) We assessed the cross-reactivity of C68.61 (upper panel) and C68.185 (lower panel) (data from Fig 2C) with 61 RBDs from sarbecovirus clades (indicated by different colors where clade 1b SARS-CoV-2-related sarbecoviruses are in blue, clade 1a SARS-CoV-1-related sarbecoviruses are in green, clade 2 ACE2-independent sarbecoviruses in yellow, clade 3 African/European sarbecoviruses in purple, and clade 4 sarbecoviruses in orange) analyzed by flow cytometry. The lower the EC50 (log10 ng/ml), the stronger binding response. Represents data from at least two independent experiments. (B) Geometric means of the EC50s (ng/ml; data from Fig 2C) and 95% confidence interval (95% CI) across clade 1b SARS-CoV-2-related sarbecoviruses and clade 1a SARS-CoV-1-related sarbecoviruses tested. (PDF) [file ppat.1012650.s004.pdf]

A

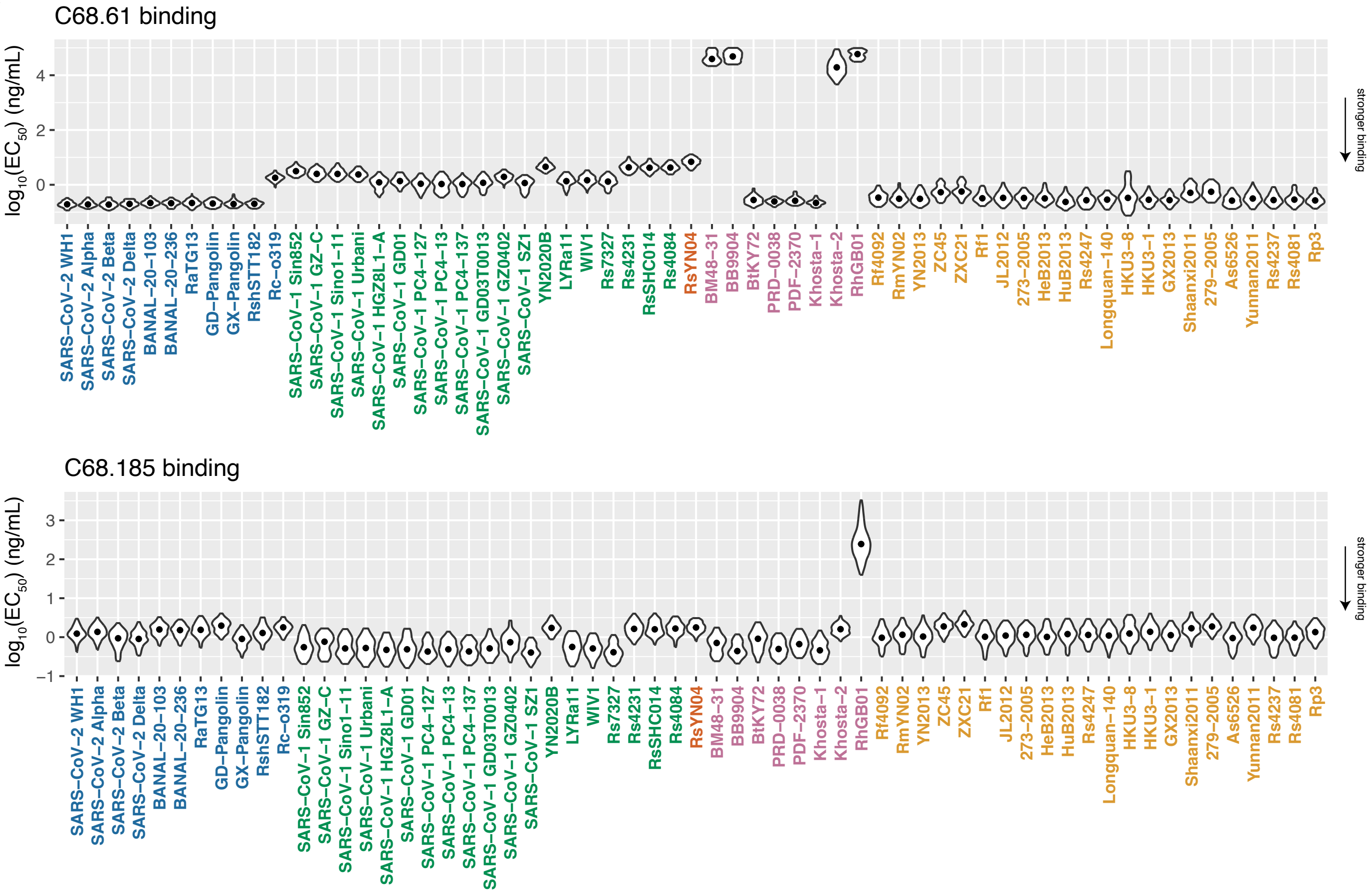

B

	**Clade 1b SARS-CoV-2 related sarbecoviruses**	
	**Geomean**	**95% CI**
**C68.61**	0.25	(0.39,0.16)
**C68.185**	1.3	(1.6,1.1)
	**Clade 1a SARS-CoV-2 related sarbecoviruses**	
	**Geomean**	**95% CI**
**C68.61**	1.9	(2.5,1.4)
**C68.185**	0.66	(0.85,0.52)
